# The Mitochondrial Foundations of Parkinson’s Disease: Therapeutic Implications

**DOI:** 10.14336/AD.2025.0440

**Published:** 2025-04-28

**Authors:** Smijin K. Soman, Micah R. J. Woodruff, Ruben K. Dagda

**Affiliations:** Department of Pharmacology, University of Nevada, Reno School of Medicine, Reno, NV 89557, USA

**Keywords:** Parkinson’s Disease;, Mitochondria, Mitophagy, Biogenesis, Mitochondrial Therapies

## Abstract

Mitochondria are dynamic organelles vital for neuronal function due to their ability to generate ATP, sequester cytosolic calcium (Ca^2+^), regulate lipid metabolism, and modulate apoptosis signaling. In order to maintain these essential functions in healthy neurons, mitochondria must be continuously replenished through mitochondrial turnover and biogenesis. Conversely, the dysregulation of mitochondrial homeostasis can lead to oxidative stress and contribute to the neuropathology of Parkinson's disease (PD). This review will provide an updated in-depth review of mitochondrial processes such as mitophagy, biogenesis, trafficking, oxidative phosphorylation, Ca^2+^ sequestration, mitochondrial transfer, and their relevance to PD pathophysiology. We provide an extensive overview of the neuroprotective molecular signaling pathways regulated by PD-associated proteins that converge at the mitochondrion. Importantly, in this review we highlight aspects of mitochondrial pathology that converge across multiple models including iPSCs, patient-derived fibroblasts, cell culture models, rodent models and chemical and genetic models of PD. Finally, we provide a comprehensive update on the molecular toolbox used to interrogate these signaling pathways using in vitro and in vivo models of PD and provide insight into the downstream protein targets that can be leveraged to develop novel therapies against PD.

## Introduction

Parkinson's disease (PD) is a progressive neuro-degenerative disease that affects dopamine-producing neurons of the *substantia nigra* (SN). A significant loss of more than 95% of midbrain dopaminergic (DA) neurons leads to the onset of motor symptoms, such as rigidity, resting tremors, and bradykinesia. While descriptions resembling PD appear in traditional Indian texts (~1000 BC) and ancient Chinese sources [1, 2], Dr. James Parkinson formally documented the clinical manifestations of PD in his published manuscript "An Essay on the Shaking Palsy" in 1817. Yet, our understanding of how SN neurons degenerate in PD is still not clear [3]. It was more than a century later (1912) until Dr. Fritz Heinrich Lewy discovered Lewy bodies in postmortem brain tissue of PD patients, which increased our understanding of PD pathology. Lewy bodies are eosinophilic, proteinaceous inclusions that progressively accumulate within neuronal soma. Biochemically, Lewy bodies are mainly composed of phosphorylated α-synuclein and phosphorylated ubiquitin. An increase in the level of Lewy bodies is associated with the neurodegeneration of SN neurons and of cortical neurons in advanced stages of PD [4]. PD is an age-related disease as patients over 65 years of age are highly susceptible to developing PD relative to younger populations. While PD is predominantly sporadic in nature (~90% of cases), 10% of cases are linked to mutations in more than 35 genes, some of which encode for mitochondrial localized proteins such as PTEN-induced Kinase 1 (PINK1). While mitochondrial dysfunction was first implicated in Parkinsonism through studies involving mitochondrial complex I inhibitors, the identification of genetic mutations in genes like PINK1, PARKIN, and DJ-1 provided definitive mechanistic evidence linking impaired mitochondrial homeostasis to the pathogenesis of PD.

Mitochondria are multi-faceted organelles that not only regulate essential neuronal functions but can also buffer calcium (Ca^2+^), regulate reactive oxygen species (ROS) signaling, can produce heat, regulate lipid and amino acid metabolism, and regulate cell fate. Unlike mitotic cells which predominantly utilize glycolysis, neurons predominantly utilize oxidative phosphorylation to survive, maintain electrical conductivity via action potentials, and establish neuronal networks. Neurons heavily rely on mitochondria for ATP production, and mitochondrial dysfunction plays a key role in the development of various neurodegenerative diseases, such as PD. It was not until 1998 that genetic mutations affecting the α-synuclein gene and subsequent accumulation of α-synuclein in Lewy bodies were associated with PD [5]. Several pathological mutations in the genes coding for PTEN-induced Kinase 1 (PINK1), Parkin, Leucine Rich Repeat Kinase 2 (LRRK2), glucocerebrosidase 1 (GBA1) and DJ-1 or PARK7 (Parkinson disease protein 7) were subsequently reported and established the genetic link to PD. At present, mutations in specific genes have been connected to particular forms of PD in more than 20 different genes, which account for 10% of all PD cases and up to approximately 5% of sporadic cases [6]. Despite the relatively lower contribution to the cumulative PD patient pool, the discovery of familial forms of PD has contributed to unraveling the mechanism behind PD development and progression. Following these landmark reports, a surge of molecular and cellular biological studies describing the physiological functions of the aforementioned gene products (PINK1, LRRK2, Parkin, DJ-1, and α -synuclein) demonstrated that these PD-associated proteins either intrinsically localize to or translocate to mitochondrion, where they regulate various aspects of mitochondrial biology in neurons. Transient expression or overexpression of PD-associated mutations in cultured primary neurons or neuronal cell lines were associated with aberrations in mitochondrial morphology (fragmentation/fission), decreased mitochondrial function (reduced oxidative phosphorylation and mitochondrial-derived ATP levels), altered mitochondrial turnover, altered mitochondrial Ca^2+^ handling, and activation of apoptosis signaling pathways [7-10]. Some of these phenotypic observations were recapitulated in knockout and transgenic mouse models, strongly suggesting that mitochondrial dysfunction plays a pathological role in PD's progression and etiology. These landmark studies gave rise to the concept that a few nuclear-encoded gene products, which show partial to complete mitochondrial localization, are critical for modulating many aspects of the life cycle and maintenance of mitochondria to provide the necessary energy essential for neuronal survival.

Considering that PD arises from multiple contributing factors, including familial environmental and age-related factors, the pathological role of mitochondria in PD is further supported by chemical models of PD. Prior to the discovery of the first set of genes that were associated with familial forms of PD, numerous cell culture and *in vivo* studies in rats demonstrated that that acute or chronic exposure to mitochondrial complex I inhibitors (6-hydroxydopamine, rotenone), that can reduce the electron flow activity from complex I to II by more than 30%, can elicit mitochondrial dysfunction and was associated with selective degeneration of SN neurons in Lewis rats [11, 12]. In *in vivo* models of PD, it was reported that rodents that were treated with complex I inhibitors tended to develop and faithfully recapitulate motor symptoms of PD including an increase in foot slips when crossing a beam balance, head tremors, and loss of gait function. As with *in vivo* genetic models of PD, Lewis rats treated with rotenone or 1-methyl-4-phenyl-1,2,3,6-tetrahydro-pyridine (MPTP) showed a progressive accumulation of Lewy bodies throughout the cortex and midbrain, progressive degeneration of SN neurons, and impaired function of brain mitochondria [13-15]. Overall, these *in vivo* studies demonstrated that both chemical and genetic models of PD converged on overt mitochondrial pathology. In brain-degenerative diseases, it is important to recognize that damaged mitochondria not only produce less ATP through oxidative phosphorylation but also display disrupted trafficking, abnormal fission and fusion processes, and impaired mitochondrial turnover, including defects in mitophagy and biogenesis. Each of these aspects of mitochondrial dysfunction is discussed in greater detail throughout this review. Furthermore, we want to emphasize the convergence in the data that have been observed across models of PD that have been employed (murine chemical and genetic models of PD, iPSC-derived neurons or human fibroblasts) to study mitochondrial-signaling pathways associated with PD pathology, as well as address some of the controversies in the field and limitations of each model. Consequently, therapies for PD designed to enhance mitochondrial function represent a promising therapeutic approach for reversing neurodegeneration linked to mitochondrial dysfunction.

## Mitochondrial Turnover in Parkinson's Disease

A fine balance between the production of functional mitochondria and the elimination of dysfunctional mitochondria is essential for maintaining fundamental cellular processes. The balance in mitochondrial turnover is attained through a synergy between two opposing cellular events, mitochondrial autophagy, termed mitophagy, and mitochondrial biogenesis. Impaired mitochondrial turnover has been implicated in the pathophysiology of PD and the targeting of these pathways is emerging as a viable therapeutic option [16, 17].

### Mitophagy

The process of orderly breakdown and renewal of cellular components (macromolecules to organelles) is called autophagy (derived from the Greek term for “self-eating”). Autophagy was first observed through electron microscopy in the form of accumulated lysosomal vacuoles in glycogen supplemented hepatocytes [18]. Autophagy serves to recycle intracellular components, enabling cells to survive extended periods of starvation by recycling biological macromolecules. However, additional roles in metabolism, immune response, and development have been reported [19]. Autophagy is a highly coordinated physiological process that is regulated by evolutionarily conserved proteins termed autophagy related proteins (ATG). Briefly, autophagy, initiated with the emergence of a membrane structure (phagophore) from the endoplasmic reticulum (ER), encapsulating segments of the cytoplasm, including the cytosol, organelles, and protein aggregates, are sequestered into a double-membrane vesicle known as an autophagosome. These autophagosomes subsequently fuse with lysosomes, where the enclosed cargo is degraded by lysosomal hydrolases. The byproducts (amino acids, nucleic acids, fatty acids) are subsequently released into the cytoplasm as macromolecules for recycling of nutrients [20, 21]. The selective removal and degradation of aged, effete, damaged, or superfluous mitochondria via autophagy is termed mitophagy [22]. Dysfunctional mitochondria as well as physiological fluctuations in cytosolic ATP levels can trigger an autophagic response culminating in the degradation of impaired mitochondria [23]. In cell culture studies, mitophagy can be induced in cells by breaching the mitochondrial membrane potential with mitochondrial uncouplers including carbonyl cyanide m-chlorophenylhydrazone (CCCP)[24].

**Figure 1.**
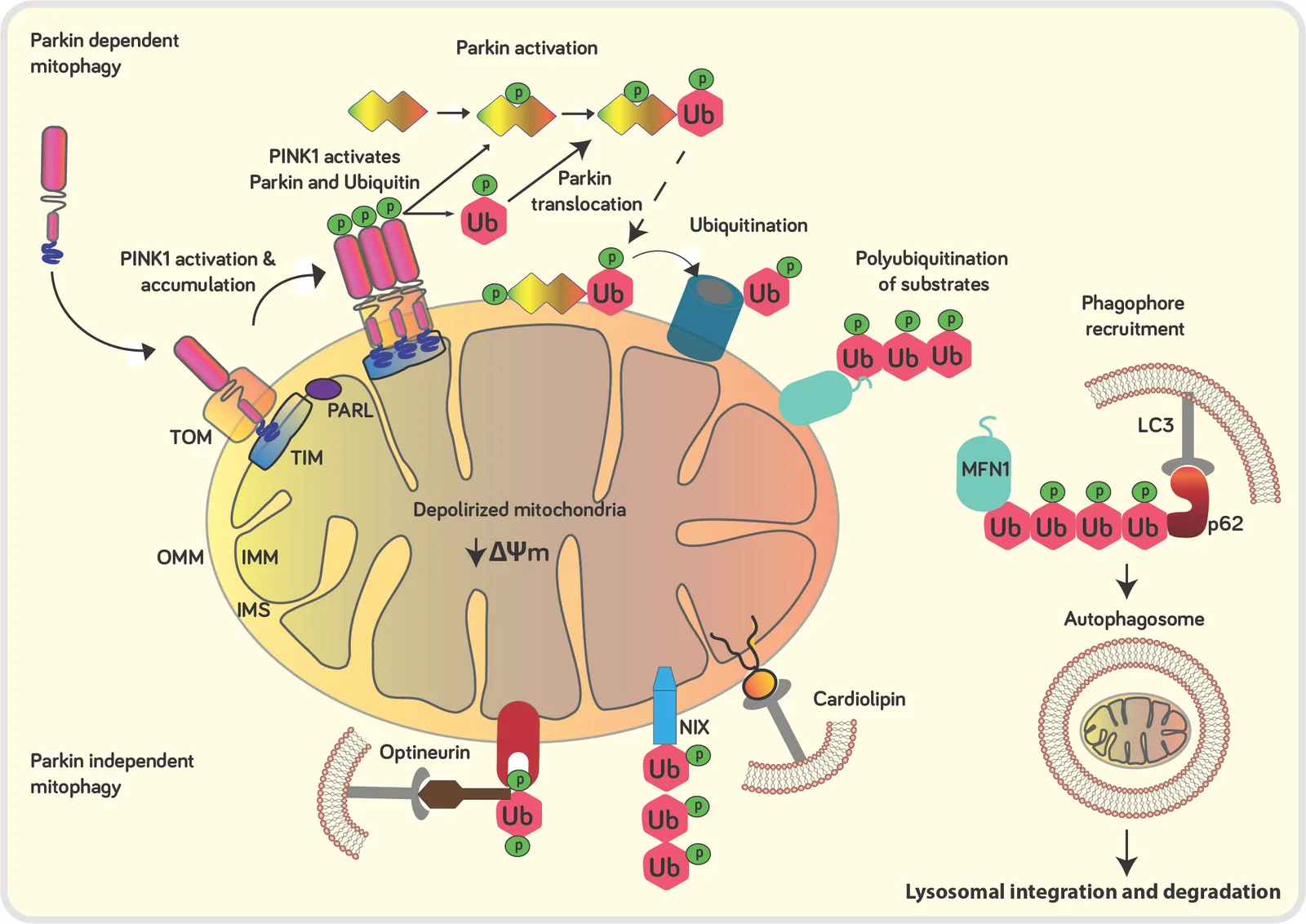
**Mechanisms of mitophagy**. In healthy neurons, most mitochondria are highly functional, as shown by a sufficient mitochondrial membrane potential that supports the import of PINK1 through the TOM-TIM protein complexes located at the outer and inner mitochondrial membranes (OMM and IMM, respectively). In functioning mitochondria, PINK1 is processed by PARL at the IMM, after which the cleaved form (cPINK1) is transported back to the cytosol, where it carries out roles outside the mitochondria, such as promoting dendrite growth, aiding neuronal development, and enhancing PI-3 kinase signaling. When mitochondrial membrane potential is lost, PINK1 import into the IMM is halted, causing its accumulation on the OMM. This buildup impairs the mitochondrial import of proteins normally mediated by the TOM complex. At the OMM, PINK1 undergoes autophosphorylation and also phosphorylates ubiquitin and Parkin, leading to the recruitment and activation of Parkin at the damaged mitochondria. Parkin then ubiquitinates various mitochondrial proteins, including VDAC1, Mfn2, and Drp1. These polyubiquitin (poly-Ub) chains are further phosphorylated by PINK1, creating a signal that attracts the autophagic machinery to initiate mitophagy. Adaptor proteins such as p62, Optineurin (OPTN), and NDP52 recognize the phosphorylated poly-Ub chains and trigger autophagosome formation by binding to LC3. Parkin-independent mitophagy involves mitophagy receptors such as NIX, BNIP3, and FUNDC1 localized to the OMM and physically bind with LC3 to mediate the elimination of mitochondria via mitophagy. Cardiolipin is externalized to OMM and interacts with autophagic receptor LC3 following impairment of mitochondrial function to flag oxidatively damaged mitochondria for downstream lysosomal-mediated degradation.

While several pathways regulate mitophagy, it is predominantly modulated via the upregulation of the PINK1-Parkin signaling pathway in response to increased oxidative stress [24, 25]. PINK1 is an atypical serine/threonine kinase that contains a mitochondria-targeting signal (MTS). Under basal conditions, PINK1 localizes to the mitochondrial membrane, and contingent upon adequate mitochondrial membrane potential (ΔΨm), it is imported through the outer mitochondrial membrane (OMM) and inner mitochondrial membrane (IMM) *via* the outer membrane (TOM) and translocase of the inner Membrane (TIM) complex respectively via its N-terminal MTS. Subsequently, PINK1 undergoes cleavage by up to four different mitochondrial processing peptidases (MPP) located in the IMM, generating a 60 kDa product. Cleavage by the rhomboid protease presenilin-associated rhomboid-like protein (PARL) and/or matrix-AAA (m-AAA) proteases results in 52/48 kDa cleaved variants of PINK1. These cleaved forms of PINK1 are then “retro-translocated” to the cytosol, where they either fulfill extra-mitochondrial functions or are degraded through the N-terminal degradation pathway [26-30]. Under these conditions, Parkin remains inactive in the cytosol. On the other hand, mitochondrial damage causes the dissolution of the ΔΨm, and un-cleaved PINK1 accumulates at the OMM bound to the translocase of the TOM complex and triggers Parkin's activation via PINK1-mediated phosphorylation of ubiquitin. While PINK1 ensures the initial mitochondrial quality check, Parkin enables the autophagic clearance of mitochondria. PINK1 phosphorylates Ser65 residues on ubiquitin and the ubiquitin-like (UBL) domain of Parkin, which activates its E3 ubiquitin ligase activity. This facilitates the ubiquitination of various mitochondrial substrates, leading to the targeted degradation of OMM proteins through the ubiquitin-proteasome pathway [31]. For instance, Parkin performs mono-ubiquitination or poly-ubiquitination of specific mitochondrial substrates (Parkin, CDC-rel1, Pael-R, synphilin-1, α-sp22, p38 tRNA synthase, cyclin E, and synaptotagmin XI) at either lysine-48 or lysine-63 linkages. The 26s proteasome can degrade individual OMM-localized proteins tagged with poly-ubiquitin chains to flag oxidatively damaged mitochondria to initiate mitophagy (Fig. 1). The addition of poly-ubiquitin chains on OMM-localized proteins electrostatically attracts ubiquitin-binding cargo receptors, including NDP52 (a.k.a. CALCOCO2), which can bind to LC3 in phagophores paving the way for the removal of damaged mitochondria [32, 33] (Fig. 1).

Several studies have highlighted the existence of signaling pathways that operate independently of PINK-Parkin based mitophagy. Some examples of autophagy receptors that physically associate with the autophagosomal membrane including Microtubule-associated protein 1A/1B-light chain 3 type II (LC3-II) and GABA Type A receptor-associated protein (GABARAP), can interact with OMM mitophagy receptors, including FKBP Prolyl Isomerase 8 (FKBP8), BCL-2 interacting protein 3 (BNIP3), NIX (a.k.a. BNIP3-like), and FUN14 domain-containing protein 1 (FUNDC1) for the selective clearance of mitochondria in the absence of Parkin [34, 35]. Additionally, IMM-localized anionic phospholipids such as oxidized cardiolipin are translocated to the OMM in response to mitochondrial damage, bind to LC3 and promote the formation of autophagosome [36]. The identification of E3 Ubiquitin ligases other than Parkin, such as Gp78 (a.k.a. AMFR), Seven in absentia homolog 1 (SIAH1) and mitochondrial E3 Ubiquitin protein ligase 1 (MUL1) in ubiquitination of mitochondrial substrates have reinforced the concept that mitophagy can occur independent of PINK1-Parkin pathway [35, 37, 38]. Dynamin-related protein 1 (Drp1)-mediated mitophagy can be activated independently of Parkin [39]. Furthermore, the activity of regulatory kinases such as Cyclin G-associated kinase (GAK) and Protein Kinase Cδ (PRKCD) play an important role in the regulation of Parkin-independent mitophagy [40].

The complexity and heterogeneity in the mitophagy pathways suggest that mechanisms that govern mitochondrial turnover have evolved to address physiological and pathological stimuli to sustain neuronal survival. In chemical and genetic models of PD, several cell culture and *in vivo* studies show that PINK1-Parkin dependent mitophagy is over-activated during the initial stages whereas other studies point to impaired mitophagy in cell culture models of chronic toxicity (rotenone and 6-OHDA) and in genetic models. For instance, neuroblastoma cells and primary cortical neurons treated with acute high doses of 6-hydroxydopamine, MPP^+^, or rotenone leads to a pathological over-activation of both macroautophagy/mitophagy leading to a reduction in mitochondria and of ATP levels [41-46]. Elevated activation of mitophagy is detrimental to neurons as transient siRNA-mediated knockdown of ATG5 and ATG7, two proteins that regulate the synthesis and remodeling of autophagosomes, increases neuronal survival against MPP^+^-mediated toxicity in cultured neuroblastoma SH-SY5Y cells [46]. *In vivo*, transgenic expression of α-synuclein is associated with mitochondrial dysfunction, leading to the aberrant accumulation of large autophagic inclusions, and loss of mitochondria; interestingly, genetic-mediated deletion of PINK1 and Parkin in α-synuclein transgenic mice (A53T) crossed with PINK1 or Parkin knockout mice worsened mitochondrial pathology, suggesting that PINK1-Parkin is cytoprotective to remove dysfunctional mitochondria [47]. Consistent with the view that PINK1 and Parkin are essential for maintaining baseline mitophagy *in vivo*, PINK1 and Parkin null Drosophila show impaired mitophagy and decreased turnover of mitochondrial proteins in the brain [48]. Although the role of PINK1 and Parkin in mitophagy in vivo remains a subject of intense debate, it is conceivable that some discrepancies may be due to the methodology used to measure mitophagy *in vivo* (the use of mito-Keima vs. mitochondrial-targeted tandem LC3 reporter (a.k.a) MitoQC as a biosensor to monitor mitophagy). Indeed, while mito-QC reporters can assay macroautophagic flux and different stages of autophagy *in vivo*, using this sensor requires rigorous analysis at multiple time points to analyze flux relative to mito-Keima which directly measures late-stage mitophagy as mito-Keima shows greater sensitivity compared to Mito-QC. While there have been conflicting reports using these two mitochondrial targeted fluorescent biosensors in terms of detecting baseline vs. PINK1-Parkin mitophagy induced by stressors, a recent study described how nuances and differences in methodology may explain contradictory reports in baseline mitophagy while providing evidence for the existence of bona fide basal PINK1-Parkin dependent mitophagy *in vivo* and in cultured cells [49-52]. In PINK1-deficient SH-SY5Y cells, a compensatory elevation in mitophagy has been noted in SH-SY5Y cells whereas other studies point to impaired mitophagy *in vivo* pointing to discrepancies between cell culture vs. in vivo genetic models of PD [25]. Consistent with this view, cell culture models that overexpress LRRK2-G2019S exhibit overactivated macroautophagy and loss of neurites in SH-SY5Y cells leading to a reduction in mitochondrial levels [47, 53], whereas LRRK2 G2019S knock-in-mice show impaired basal mitophagy that contributes to PD pathology [54].

Regardless of whether mitophagy is impaired or over-activated, tilting the balance either way is detrimental as dysfunctional mitochondria (e.g. uncoupled mitochondria, reduced ox/phos, high ROS) eventually accumulate leading to an increase in oxidative stress. However, impairment in mitophagy signaling pathways seem to be a more common pathological theme in PD causing the build-up of dysfunctional mitochondria and contributing to the pathology of PD. Fibroblasts derived from human subjects harboring Parkin mutations exhibit increased mitochondrial dysfunction, as evidenced by decreased complex 1 activity, decreased transmembrane potential, impaired ATP synthesis and increased ROS levels [55]. PD patients harboring homozygous mutations in PINK1, including a family of Italian descent, showed symptoms clinically identical to idiopathic PD patients [56, 57]. In addition to phosphorylating direct effectors of mitophagy pathway such as Parkin and Ubiquitin, PINK1 also phosphorylates substrates like Protein Kinase A (PKA) and Parkin Interactive Substrate (PARIS), regulating extramitochondrial functions [58, 59]. This extra-mitochondrial function implicates a broader role of PINK1 in neuronal health. The ATP analog, kinetin triphosphate which pharmacologically activates PINK1, restores the kinase activity of a PD-associated mutant of PINK1 and concomitantly elevating the kinase activity of endogenous wild-type PINK1, uncovering a potential new approach for therapeutically targeting the PINK1/Parkin pathway [60]. Recently, a study provided evidence that treatment with cytarabine, a marine-derived compound used to treat leukemia, activates AMP-activated protein kinase (AMPK), a signaling protein involved in mitophagy and mitochondrial biogenesis. Additional cell culture studies in HEK293T cells showed that AMPK activation subsequently increased the protein levels of mitophagy-related proteins Parkin, PINK1, and DJ-1 to exert neuroprotective effects [61-63]. Another study further supported the role of AMPK in the regulation of mitophagy during fluoride induced developmental neurotoxicity in cell culture models and in rats treated with NaF [64]. While there is convergence across murine and human-derived models of PD indicating an upregulation of macroautophagy and mitophagy, it is worth noting that these data is congruent with several studies conducted in postmortem brain tissue showing increased levels of LC3-II which likely reflects impaired macroautophagy in idiopathic cases of PD, but not in familial case of a PD human harboring the LRRK2-G2019S mutation [65].

### Mitochondrial Biogenesis

In conjunction with mitophagy, mitochondrial biogenesis is not only critical for the replenishment of aged mitochondria with healthy mitochondria but is necessary to establish a minimum level of functional mitochondria necessary for maintaining and consolidating dendritic networks and axons, mitochondrial biogenesis is triggered by external stimuli and stress signals. For instance, both nutritional deficiencies and exercise can increase mitochondrial biogenesis in cells. The field of mitochondrial biogenesis research emerged from the observations made by Dr. John O. Holloszy in his experiments with mice, where he saw a 60% increase in total protein content in the mitochondrial fraction of muscle tissue from mice subjected to strenuous exercise, confirming that mitochondrial content can be regulated based on energy demands [66]. The mitochondrial genome, a circular DNA molecular of 16.5kb, encodes for a few membrane proteins (13) of the oxidative phosphorylation machinery, ribosomal RNAs and tRNAs [67]. The rest of the 1000-1500 proteins needed (~99%) for mitochondrial function, including mtDNA replication and expression, are encoded in the nuclear genome, produced by cytosolic ribosomes as precursor proteins, and subsequently imported into the mitochondria [68].

Like mitophagy, mitochondrial biogenesis is a intricate intracellular process, which upon demand, increases the availability of mitochondrial proteins through a multi-step process involving mtDNA replication, mtDNA transcription, translation of nuclear-encoded transcripts of mitochondrial-targeted proteins, and import of nuclear-encoded mitochondrial-targeted proteins across the mitochondrial membrane, leading to an enriched mitochondrial network [69, 70]. This intricate, multi-step process is mainly regulated by the nuclear-encoded peroxisome proliferator-activated receptor-γ coactivator (PGC)-1α and the mitochondrial transcription factor A (TFAM). PGC-1α acts as a transcriptional co-activator for nuclear respiratory factors (NRFs), which, together with TFAM, enhance transcription of multiple mitochondrial genes responsible for encoding ATP synthase, cytochrome c, cytochrome c oxidase IV, and TFAM [71]. TFAM translocates to the mitochondrial matrix and stimulates mtDNA replication and mitochondrial gene expression [72]. Parkin, in addition to its role in mitophagy, has also been shown to associate with TFAM to enhance mitochondrial transcription and replication [73]. The expression of PGC-1α is modulated by various signaling pathways, including the cAMP/PKA/cAMP response element-binding protein (CREB), p38 mitogen-activated protein kinase (p38MAPK), and AMPK [63, 74]. Specifically, after mitochondrial damage, AMPK signals mitochondrial degradation via activation of FNIP1 and transcription factor EB (TFEB) prior to enhancing mitochondrial biogenesis by elevating the expression of PGC-1α and estrogen-related receptor alpha (ERRα) [63]. Furthermore, environmental and various physiological stimuli initiate mitochondrial biogenesis. Both exercise and caloric restriction have been shown to enhance the expression of genes such as TFAM, PGC-1α, eNOS, and SIRT1, leading to increased mitochondrial volume [75, 76]. Similarly, exercise was shown to upregulate PGC-1α and Brain-derived neurotrophic factor (BDNF) in mice; this cleaved form of BDNF enhances CREB-dependent PGC-1α expression to stimulate mitochondrial biogenesis and support hippocampal synapse formation and maintenance in vivo [77-80]. Oxidative stress is another significant stimulus for mitochondrial biogenesis. An elevation in localized concentration of superoxide, generated in response to the inefficient electron flow in complexes I and IV as a consequence of elevated mitochondrial respiration or dysfunction, can elicit mitochondrial biogenesis by increasing the protein levels of PGC-1α [81].

Impaired mitochondrial biogenesis, in conjunction with mitochondrial dysfunction and reduced mitophagy, are factors that play a prominent pathological role in the etiology of PD. It is important to highlight that these aspects of mitochondrial function are equally impaired by aging, suggesting that aging can exacerbate mitochondrial dysfunction in PD. In support of this concept, a reduced mtDNA copy number and an increase in the mutational burden of mtDNA, are potential biomarkers of PD as patients harboring mutations in mitochondrial DNA polymerase (POLG) reported to show Levodopa-responsive PD symptoms. Additionally, rodent experimental models have shown that mutations in POLG can cause significant degeneration of DA neurons in the SN pars compacta (SNpc) but not of other areas, including the ventral tegmental area [82-84]. It is worth noting that mutator mice, that express a defective POLG, accelerate the accumulation of mtDNA mutations with age but show no visible loss of DA neurons, underscoring the role of aging in enhancing mitochondrial dysfunction. On the other hand, mutator mice that lacked endogenous Parkin exhibit overt neurodegeneration and Levodopa-responsive Parkinsonism [85]. Alteration of mitochondrial biogenesis via downregulated PGC-1α expression is observed in idiopathic PD patients [86]. The observation that mitochondrial biogenesis is impaired in idiopathic PD is also seen in familial PD (PD-associated mutations in PINK1, DJ-1, and Parkin). A decrease in mitochondrial biogenesis has been observed in cell culture and *in vivo* models of PD. Specifically, a conditional knockout loss of Parkin inhibits mitochondrial biogenesis by promoting the accumulation of the PARIS, leading to the suppression of PGC-1α expression in mice. [87]. In another study, pharmacological induction of mitochondrial biogenesis by using 5-hydroxytryptamine (5-HT1F) receptor agonist-LY344864 attenuated DA neuron loss in 6-OHDA based rodent PD model. Therefore, stimulation of mitochondrial biogenesis appears to be a promising therapeutic target for treating PD [88]. While there have not been many studies conducted in iPSC-derived midbrain neurons that have examined the status of mitochondrial biogenesis compared to patient-derived fibroblasts, it is worth noting that a similar reduction in mitochondrial biogenesis - as evidenced by a significant reduction in mitochondrial content and PGC-1α mediated mitochondrial biogenesis, has been observed in Parkin-deficient iPSC-derived midbrain DA neurons from PD patients compared to their respective isogenic controls [89]. However, it is also worth emphasizing that in addition to a decrease in the protein levels of TFAM, PGC-1α as well as the level of several proteins in mitochondria have been shown to be reduced in the SN and striatum of postmortem PD tissue which is consistent murine and human-derived models of PD, and thereby underscores the importance of analyzing mitochondrial biogenesis-related signaling pathways in multiple complementary PD models [90].

## Mitochondrial dynamics and trafficking in Parkinson's Disease

### Mitochondrial Dynamics

Mitochondrial fission and fusion (MFF) processes is a well-coordinated, physiological process regulated by large GTPases from the dynamin superfamily, which are localized at the OMM or IMM. [91]. MFF modulators are capable of shaping mitochondria into spherical, tubular, or highly interconnected networks. Mitochondrial fission occurs via the translocation of Drp1 from the cytosolic compartment to Fis1 bound at the OMM. Other mitochondrial fission proteins include mitochondrial fission factor (Mff), MiD49 and MiD51 (Mitochondrial Dynamics proteins of 49 kDa and 51 kDa, respectively) [92]. Drp1 is a GTPase mechanoenzyme that assembles into oligomeric rings, which constrict the mitochondrion, resulting in the division of the mitochondrion into two "daughter" mitochondria. Drp1 has a cardiolipin binding motif that associates with cardiolipin that has externalized onto the OMM due to cellular stress [93]. Mitochondrial fusion is facilitated by another group of GTPase mechanoenzymes, such as Mitofusin 1 and 2 (MFN1/2), which drive the fusion of the outer mitochondrial membrane (OMM) bilayers, while Opa1 governs the fusion of the inner mitochondrial membrane (IMM) bilayers, completing the mitochondrial fusion process [94-96]. Similar to Drp1, MFN1/2 are form homo- and hetero-oligomeric rings to facilitate mitochondrial fusion [92]. Recently, MFN1 has been shown to interact with MTCH2 to sustain mitochondrial fusion alongside MFN2 [97]. Treatment with the small molecule agonist of MFN1, S89, has been shown to enhance mitochondrial fusion to rescue mitochondrial dysfunction [98]. The MFF machinery is posttranslationally regulated by phosphorylation, ubiquitination, sumoylation, and O-GlcNacylation of proteins located on the OMM. For instance, the mitochondrial fission activity of Drp1 is reversibly regulated via direct phosphorylation by PKA on Ser 637, which impairs its GTPase activity and dephosphorylated by the phosphatase calcineurin at the residue to elevate its mitochondrial-specific fission activity [99, 100].

Mitochondrial dynamics governed by the MFF machinery is essential for neuronal survival and “healthy” brain aging. Excessive mitochondrial fission, induced by enhanced mitochondrial fission activity of Drp1, makes neurons susceptible to apoptosis [92]. Overt Drp1-mediated fission is associated with increased neurodegeneration of cultured hippocampal and cortical neurons induced by several stressors, including hydrogen peroxide, rotenone, 6-hydroxydopamine, and other stressors [43, 101, 102]. On the other hand, enhanced mitochondrial fusion by the pro-fusion regulators protects neurons against neuronal apoptosis induced by excitotoxicity, oxidative stress or against mtDNA mutations induced by aging [103, 104]. Treating primary cortical neurons with rotenone induces overt mitochondrial fission, an event that is upstream of mitochondrial dysfunction, oxidation, and translocation of cardiolipin from the IMM to the OMM, and eventual degradation of mitochondria via the PINK1-Parkin pathway [36]. Linking Drp1-mediated mitochondrial fission to neurodegeneration in models of PD, small molecular compounds that block the activity of Drp1 (e.g., Mdv1) can block neurodegeneration induced by complex I inhibitors (rotenone), thereby blocking neuronal death in rotenone treated rats [105]. Consistent with chemical models of PD, genetic models of PD have provided evidence that aberrant Drp1 mediates mitochondrial fission which contributes to neurodegeneration. PINK1 and DJ-1 are mitochondrial neuroprotective proteins, as loss of endogenous PINK1 or DJ-1 sensitizes neurons to Drp1-dependent apoptosis induced by cell stressors, whereas transient or stable expression of PINK1 or of DJ-1 promotes mitochondrial fusion and protects neurons from oxidative stress (H_2_O_2_, MG132, staurosporine) [25, 56, 106-110]. In cultured PINK1-deficient neurons, enhanced mitochondrial fission and loss of mitochondrial integrity occurs in part via a decrease in PKA-modulated phosphorylation of Drp1, along with a concurrent increase in activity of PP2A/calcineurin[111]. Recently, a research report showed that mitochondrial fusion in primary cortical neurons can be enhanced with the treatment of exogenous, recombinant BDNF through PKA-mediated phosphorylation of Drp1. In addition, treatment of primary cortical neurons with exogenous human BDNF reduced the fission of dendritic mitochondria and prevented the loss of dendritic mitochondria induced by the complex I inhibitor rotenone [98]. Therefore, phosphorylation of Drp1 and its subsequent deactivation via PKA inhibits mitochondrial fission thereby supporting neuronal health and functioning at least as demonstrated in cell culture models [80, 112]. PINK1, Parkin, and DJ-1 have been observed to form a trimeric complex in the mitochondria to regulate the ubiquitination of Parkin itself and Parkin substrates, including synphilin-1 [113]. This trimeric complex is critical for governing mitochondrial structure and function [114]. While transient expression of PINK1 can rescue the lack of mitochondrial mobility and fragmentation in DJ-1 deficient cells, transient expression of Parkin can reverse mitochondrial pathology and neurodegeneration of SN neurons in PINK1 null Drosophila models but not by enhanced expression of DJ-1 [115].

Overall, these epistasis studies in cell culture models suggest that Parkin is positioned downstream of PINK1 while DJ-1 is positioned upstream of both PINK1 and Parkin [115-117]. In healthy cells, Parkin interacts with PINK1 to maintain mitochondrial integrity and morphology. Specifically, Parkin elicits the ubiquitination of Mff in a PINK1-dependent manner and via another e3 ubiquitin ligase [118]. Beyond forming a heterotrimeric complex, there is some experimental evidence that DJ-1 and PINK1 can reversibly interact with the catalytic subunit of PKA to govern mitochondrial function and dynamics. In support of this concept, an in vitro pull-down study suggests that PKA/C can phosphorylate DJ-1 at T154 to enhance the dimerization of DJ-1, a critical molecular event required for the antioxidant/ neuroprotective activity of DJ-1 in neurons [119]. Vacuolar protein sorting 35 (VPS35), a PD-associated gene encoding for a key protein facilitating the recycling of endosomal proteins, skews the fine balance toward excessive fission through enhancing mitochondrial fragmentation and dysfunction via increased DLP1 (yeast homologue of Drp1) turnover of mitochondrial-derived vesicles (MDV) [120-123]. As in neuronal cell lines and cultured primary neurons, emerging data in iPSC-derived dopamine neurons that harbor different mutants of PINK1, or LRRK2 recapitulate alterations in the MFF machinery as evidenced by overt mitochondrial fission and decreased phosphorylation of Drp1 compared to isogenic controls [124]. In Parkin null (-/-) iPSCs-derived DA neurons, smaller mitochondria with a swollen phenotype (e.g. loss of matrix density), accompanied by a decrease in mitochondrial content and mitochondrial volume was noted in Parkin deficient (-/-) iPSCs-derived DA neurons [125]. Another study in iPSC-derived DA neurons showed similar mitochondrial swelling phenotype in two patients harboring a PINK1 (PINK1-Q456X) or Parkin mutation (V324A) that had a concomitant increase in mitochondrial superoxide levels. These differences in mitochondrial morphology associated in iPSC-derived DA neurons may be attributed to differences in the time points analyzed in culture (2 months vs. > 3 months) which suggests that while mitochondrial fission may be initially observed, mitochondrial swelling may occur in long term cultures (>3 months); although this mitochondrial phenotype is interesting, it is not recapitulated across models of PD (neuronal cells, primary neurons vs. iPSC-derived neurons). However, it is worth noting mitochondrial swelling is recapitulated by rotenone or 6-OHDA treatment of primary neurons at high doses, but not at lower doses, which may be caused by an increase in the activation of the mitochondrial permeability transition pore (MPT) [126] and an increase influx of Ca^2+^ as evident in rat brain mitochondria, SH-SY5Y neuroblastoma cells and in PC12 cells [127]. Consistent with the concept that mitochondrial fragmentation contributes to mitochondrial pathology, it is worth noting that a decrease in the protein levels of Drp1 has been reported in the SN, caudate and putamen of postmortem brain tissue from PD humans, data that is highly congruent with mouse models of PD [65], which underscores the need to use more than one complementary model of PD to study signaling pathways that regulate mitochondrial dynamics in the context of PD. The implication of these proteins in mitochondrial dynamics gives insight into the pathophysiology of PD.

### Mitochondrial trafficking and extracellular transfer

Although mitochondrial movement has been documented for many years, advances in fluorescent labeling and live imaging have given insight into the mechanisms governing mitochondrial trafficking [128-130]. Mitochondria are dynamic organelles that are transported to distal ends of neuronal processes like axons and dendrites for localized and targeted functions such as providing ATP and sequestering Ca^2+^ within the microdomains of a neuron [131]. Additionally, dysfunctional mitochondria must be removed and delivered to autophagosomes for mitophagy. Mitochondrial trafficking is vital for neuronal function, as evidenced by studies in ric-7 mutant *C. elegans,* where spontaneous neuronal degeneration occurs due to impaired mitochondrial trafficking [132]. Consequently, erroneous mitochondrial transport and clearance can be detrimental to neuronal function and have been associated with several neurological disorders [133-136].

Mitochondrial movement is heterogeneous and bidirectional in nature. Based on the polarity of the cell, mitochondrial movement is classified into anterograde and retrograde movement. An intricate mechanism constituting the adaptor/motor protein complex and cytoskeleton proteins operates the movement of mitochondria. Anterograde movement toward the (+) end of microtubules is facilitated by kinesin motors, which form complexes with cargo and adaptor proteins. The kinesin superfamily proteins (KIFs) are large ATPases characterized by a globular N-terminal motor domain that attaches to microtubules and harbors a C-terminal domain that connects to cargo or adaptor proteins associated with organelles, such as mitochondria. Trafficking kinesin-binding proteins 1 (TRAK1) and 2 (TRAK2) serve as adaptors, interacting with kinesin to regulate mitochondrial motility [137]. The motor/adaptor complex needs a mitochondrial receptor in the form of Miro1 for attachment to mitochondria [138]. Mitochondrial Rho GTPase 1 (Miro1) and Miro2 are GTPases with a pair of EF-hand Ca^2+^ binding domains and harbor a C-terminal transmembrane (TM) domain that acts as an anchor to the mitochondria [139, 140]. These Miro GTPases act as key mediators of intracellular transport of mitochondria [141]. On the other hand, the retrograde movement towards the (-) end of the microtubules is facilitated by cytoplasmic dynein. Dynein consists of two heavy chains that function as motor proteins and associate with microtubules, along with other proteins (intermediate chains, light intermediate chains, and light chains) that modulate its function and cargo attachment.

**Figure 2.**
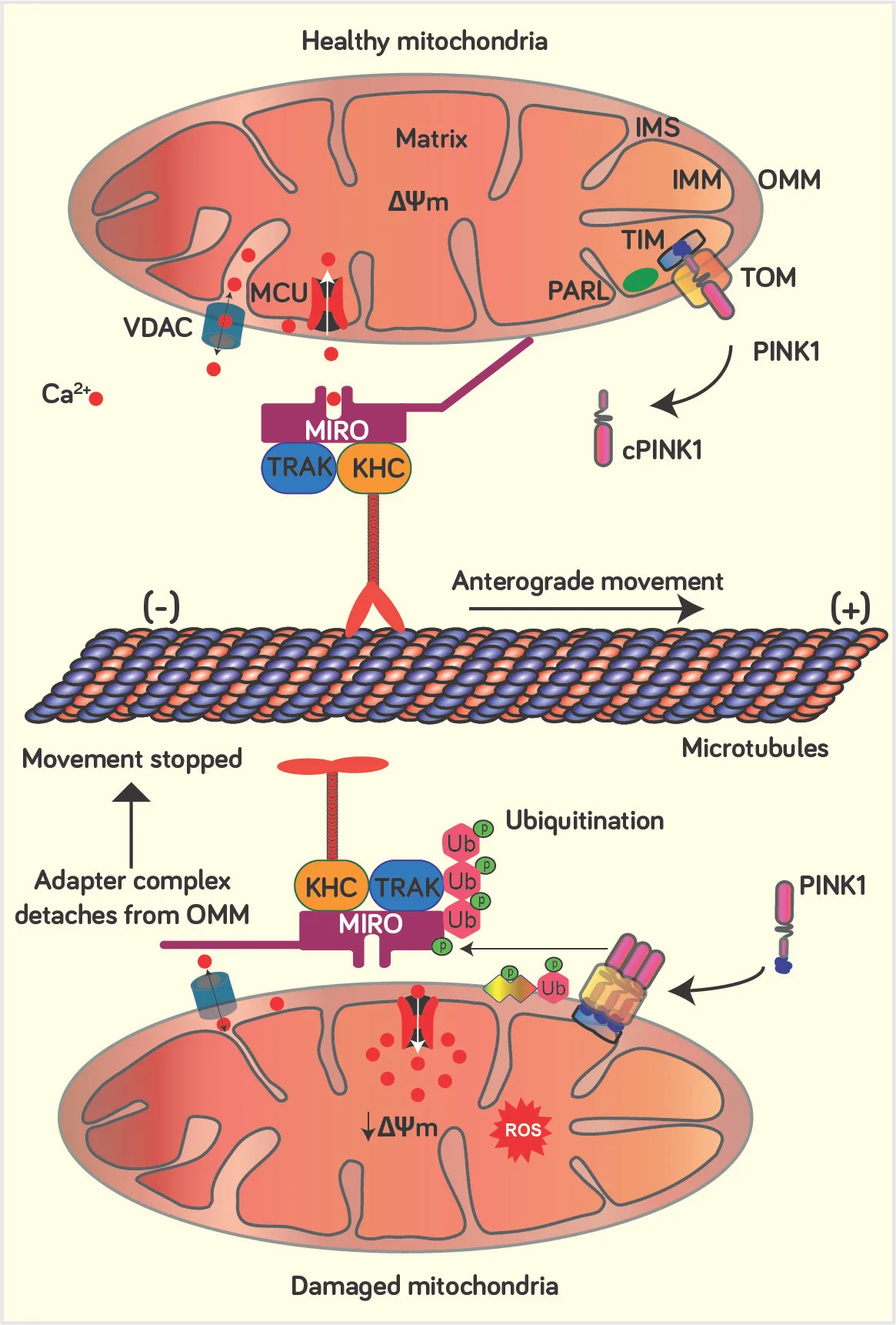
**Altered mitochondrial trafficking in PD contributes to PD pathology**. Mitochondrial trafficking machinery involves a motor/adaptor complex attached to microtubules. Under physiological conditions, healthy neurons can mobilize mitochondria towards distal sections of dendrites and axons via anterograde or retrograde trafficking by utilizing molecular adaptors termed the mitochondrial transport complexes, which anchor mitochondria to microtubules. Under healthy conditions, PINK1 is intrinsically targeted to the OMM via its MTS localized at the N-terminal domain, where it is proteolytically cleaved by intermembrane space and IMM-localized proteases upon gaining entry inside the mitochondrion. Cleaved PINK1 (cPINK1) gets shuttled to the cytoplasmic compartment to govern extra-mitochondrial functions, including anterograde trafficking in dendrites or stalling mitochondrial at distal ends of axons. In axons, the Miro-Milton (or Miro1/2-Trak) adaptor complex drives KIF5-driven anterograde mitochondrial movement. PINK1 and Parkin associate with Miro on depolarized mitochondria to stall mitochondria which facilitates their autophagic-mediated sequestration as phosphorylation of Miro1 by PINK1 facilitates proteasomal degradation of Miro1 via a Parkin-dependent mechanism. Proteolytic degradation of Miro uncouples damaged mitochondria from the microtubule network. In PD, SN neurons cannot reduce PINK-Parkin mediated stalling of oxidatively-damaged mitochondria leading to an overt buildup of uncoupled mitochondria in axons that promote neurodegeneration by extruding ROS.

Mitochondrial movement is primarily dictated by various intercellular and intracellular events that demand energy or Ca^2+^ sequestration [142]. Immobile mitochondria are usually stationed at the site of high energy demand like the axoneme of sperm, where motor proteins drive motility of sperm [143]. Neurons are energy-intensive and mitochondrial recruitment is increased during periods of high neuronal activity and synaptic plasticity. Mitochondria synthesize ATP and regulate Ca^2+^ levels to enable and govern synaptic transmission. Miro1, through its EF-hand Ca^2+^ binding domain, senses elevated Ca^2+^ levels and undergoes conformational changes leading to the uncoupling of the Kinesin-TRAK-Miro transport machinery and subsequent inactivation. Additionally, elevated levels of Ca^2+^ have been shown to inhibit MFN through Miro1, thereby decreasing mitochondrial fusion [144]. Removing mitochondria from axon terminals leads to impaired synaptic transmission, likely caused by an inadequate ATP supply or disrupted Ca^2+^ transients [145]. In axons, PINK1-Parkin signaling axis regulates Miro turnover. PINK1 phosphorylates Miro1, resulting in Parkin-mediated ubiquitination and subsequent arrest of mitochondrial transport [146] (Fig. 2). Evidence for the targeting of Miro1 as a therapeutic approach for a subset of phenotypes in PD has shown promise [147].

Trafficking of mitochondria is also influenced by BDNF signaling. BDNF has been demonstrated to increase mitochondrial presynaptic localization through Miro1-Ca^2+^ signaling. Furthermore, a recent study showed that cultured cortical neurons treated with recombinant human cleaved BDNF significantly increased bidirectional trafficking of mitochondria in dendrites through PKA-mediated phosphorylation of Miro1, a physiological event that was associated with an increase in the level of dendritic mitochondria [80, 112, 148]. Mitochondrial transport is vital for sustaining crucial cellular mechanisms such as mitophagy. Pathological mitochondrial insult is often accompanied with impaired mitochondrial trafficking. Miro1 expression is disrupted in fibroblasts derived from individuals with PD carrying Parkin mutations [140]. Anterograde mitochondrial movement is disrupted in murine DA neurons deficient in respiratory chain subunits [149]. PINK1-facilitated phosphorylation of Miro1 impairs mitochondrial transport, and PINK1-depleted primary cortical neurons exhibit impaired mitochondrial movement [150]. Moreover, LRRK2 regulates timely degradation of Miro1, and pathogenic LRRK2-G2019S disrupts mitochondrial trafficking and delays mitophagy [145, 151]. Impaired mitochondrial mobility disrupts the proper distribution of mitochondria, leading to an increased accumulation of potentially dysfunctional mitochondria. In iPSC-derived DA neurons, several studies performed in PD-patient derived fibroblasts harboring mutations in oligomerization-prone mutant α-synuclein (E57K) that were converted TH-positive neurons, or that express a mutant of Miro1 (R272Q) showed significantly impaired baseline or reduced anterograde mitochondrial trafficking along with decreased expression of Miro1 [152, 153]. Other iPSC-derived DA neurons expressing a LRRK2 mutant (G2019S) showed an inability to stop mitochondria in axons to allow for the clearance of Miro1 via mitophagy. However, it is worth noting that while the number of studies analyzing mitochondrial trafficking in iPSC-derived DA neurons from PD patients have been limited, emerging evidence shows some congruency and convergence with murine models of PD.

In addition to intracellular mitochondrial trafficking, cells can exchange organelles and other cellular components. Intercellular mitochondrial transfer, also known as horizontal mitochondrial transfer, is a process where recipient cells obtain mitochondria from donor cells to restore their respiratory function and improve survival. This well-described bona fide physiological process contributes towards enhancing mtDNA content and functional recovery of recipient cells [154-156]. Several studies have posited several plausible mechanisms that occur in neurons for mediating intercellular mitochondrial transfer. Tunneling nanotubes (TNT) can form between two cells to facilitate the transfer of organelles, including mitochondria, and other cellular components. TNTs originate from stressed cells due to the involvement of p53, a key protein in apoptosis signaling [155, 157, 158]. Oxidative stress in neurons drives the upregulation of p53 which causes the cleavage of the protein S100A4 by caspase-3. The concentration of extracellular S100A4 guides the formation of TNTs through its interactions with its cognate receptor: Receptor for Advanced Glycation End Product (RAGE). Recipient cells, in comparison to the donor cell, have a higher concentration of extracellular S100A4 which drives TNT formation and connection between healthy and stressed cells. Astrocytes can function as both recipient and donor cells in the formation of TNTs, while neurons can form TNTs with astrocytes in an activity-dependent manner [156, 158]. Interestingly, despite the potential benefits afforded by transferring mitochondria between cells. Evidence has shown that TNTs also enable rapid transfer of α-synuclein between cells, possibly enhancing the pathology of PD [159]. Conversely, the intercellular transfer between astrocytes via TNTs of mitochondria to α-synuclein burdened cells and α-synuclein to healthy cells may have protective effects [160]. Extracellular vesicles (EVs) are another mode of transfer that can move whole mitochondria or mitochondrial components, such as mtDNA, proteins, or cardiolipin, between cells [161, 162]. A study showed that synaptosomes, an artificial subcellular fraction of synaptic terminal components, were found to be viable vehicles for mitochondrial transplantation *in vitro* [163]. Additionally, gap junctions can mediate the exchange of mitochondria. Specifically, in postmortem brain tissue from PD humans, Connexin 43 (Cx43) can mediate the transfer of mitochondria for protection from ischemia/reperfusion injury [164, 165]. Recently, in post-mortem late-stage PD brain tissue, Cx43 was found to be at significantly reduced levels across several brain regions [166]. These observations could suggest a connection between hindered extracellular mitochondrial transfer and PD pathology, though further investigation needs to be carried out to better understand this association. The physiological role of mitochondrial transfer in PD is still yet unknown. However, research in these areas offers a new strategy for addressing mitochondrial dysfunction in PD.

## Mitochondrial Respiration and Ca^2+^ Homeostasis in PD

Alteration to mitochondrial function and homeostasis has a large impact on the energetics of the cell. A disruption in mitochondrial function plays a prominent role in contributing to neurodegeneration of midbrain DA neurons in PD. Indeed, a significant decrease in the activities of complex I and complex IV, a reduction in the activity of citric acid cycle (TCA) enzymes, and a decrease in mitochondrial-derived ATP synthesis are bioenergetic alterations that occur in response to mitochondrial dysfunction in PD. Similarly, maintenance of Ca^2+^ homeostasis within mitochondria is essential to the function of the organelle and the health of the cell at large. Mitochondrial Ca^2+^ overloads in PD because of dysregulation has a drastic impact on the viability of these essential organelles.

**Figure 3.**
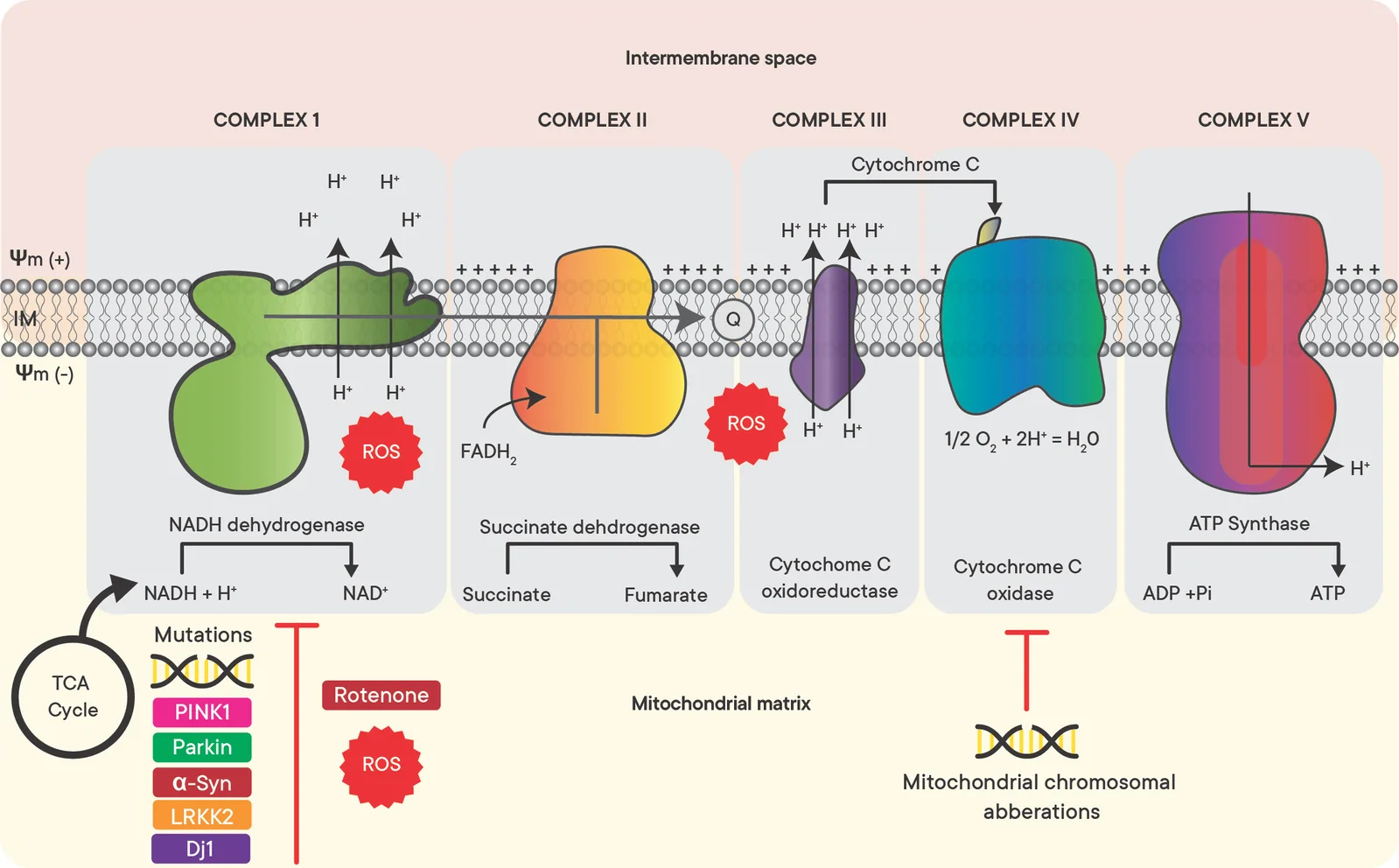
**Regulation of mitochondrial oxidative phosphorylation by PD-associated proteins**. The five multimeric ETC complexes localized at the IMM include 1) NADH dehydrogenase complex (complex I), composed of 46 subunits that mediate the oxidation of electron carriers NADH to NAD+, 2) succinate dehydrogenase (complex II), which consists of four subunits that contain the cofactor FAD and accepts succinate as the substrate which is catabolized to fumarate, 3) cytochrome C oxidoreductase (cytochrome c reductase, complex III) consists of an iron-sulfur complex of 11 protein subunits and 3 cytochromes, 4) cytochrome C oxidase (complex IV) is a heme-containing complex comprised of 13 subunits, and ATP synthase (complex V). ATP synthase is a large mushroom-shaped multimeric enzyme composed of a channel embedded in the IMM (F0 sector), which accepts protons, and a stalk structure that protrudes out of the IMM that catalyzes the conversion of ADP to ATP (F1 sector). Before the generation of ATP by complex V, oxygen accepts a pair of electrons to form H2O through an enzymatic process regulated by complex IV. By establishing an electrochemical gradient, the high concentration of protons accumulated in the intermembrane space allows for the flow of protons through ATP synthase, going down the potential energy gradient necessary to produce the mechanical energy to rotate the F1 portion of the ATP synthase in a counterclockwise rotation manner. In this manner, three protons that go through the canal of the Fo portion of the ATP synthase generate 1 molecule of ATP per cycle by covalently coupling a phosphate to ADP. During the progression of PD, SN neurons experience a substantial increase in the intramitochondrial level of ROS (e.g., superoxide) because of the backflow of electrons that occur predominantly in complex I as well as complex II, leading to an overt accumulation of mutations in the mitochondrial genome (mtDNA), a decline in ATP levels, and a reduction in transmembrane potential. The PD-associated gene products that show partial localization to the mitochondrion and are shown to regulate oxidative phosphorylation by regulating different ETC complexes (e.g., complex 1) or ATP synthase include PINK1, Parkin, DJ-1, and LRRK2. PD-associated mutations in these proteins decrease complex I and IV activities, reduce transmembrane potential, decrease ATP synthesis, reduce the activity of TCA enzymes, and decrease proper assembly of ETC complexes.

### Mitochondrial Respiration

Aerobic respiration is a critical physiological process that begins with the catabolic breakdown of glucose through glycolysis, the tricarboxylic acid (TCA) cycle, and the electron transport chain (ETC). Oxidative phosphorylation is driven by a large multimeric ETC protein complex (I-IV) localized in the inner mitochondrial membrane (IMM), facilitating ATP production through the ATP synthase enzyme (Complex V). Only complexes I, III, and IV actively pump protons from the mitochondrial matrix into the intermembrane space, establishing an electrochemical gradient that powers ATP synthesis by Complex V (Fig. 3). The concept that mitochondrial dysfunction contributes to PD etiology was evident when a clinical study in 1983 reported that four individuals intoxicated with the illicit use of a drug containing contaminated amounts of the complex I inhibitor 1-methyl-4-phenyl-1,2,5,6-tetrahydropyridine (MPTP) laced with minute quantities of 1-methyl-4-phenyl-4-propionoxy-piperidine (MPPP) developed severe forms of Parkinsonism [167]. This landmark study gave rise to the concept that mitochondrial toxins inhibiting the activity of mitochondrial complex I contribute to the selective neurodegeneration of SN neurons in PD [168]. In further support of this concept, several *in vitro* studies showed that primary neurons and DA neuronal cell lines treated with neurotoxins MPP+, the active byproduct of MPTP, or 6-OHDA exhibited a significant decrease in complex I and IV activities, a reduction in transmembrane potential, and a decrease in mitochondrial-derived ATP synthesis before undergoing neurodegeneration [13, 14, 169, 170]. Recently, complex I deficiency has been identified to be a delineating factor between subtypes of idiopathic PD [171]. Disruptions in complex I activity can alter the oxidative environment within mitochondria and increase reactive oxygen species prevalence. Recent studies have demonstrated significantly lower levels of reduced glutathione, a key antioxidant, in postmortem samples of the SN of PD patients. Studies have provided evidence that treatment with polyphenols, a plant antioxidant, has therapeutic advantages in PD [172-174]. BDNF has also been shown to enhance mitochondrial respiration through complex I [175, 176]. In a recent study, exogenous treatment of murine brain derived isolated mitochondria with exogenous BDNF increased oxygen consumption, as evidenced by an increase in oxidative phosphorylation [112]. Hence, given that the mRNA and protein level of BDNF is significantly reduced in the substantia nigra of postmortem brain tissue obtained from PD humans [65, 177], targeting the BDNF signaling pathway has emerged as a potential therapeutic option to enhance mitochondrial respiration [80, 178, 179]. Previous cell culture and *in vivo* studies that analyzed the impact of PD-associated mutants of PINK1, LRRK2, DJ-1, and α-synuclein have provided mechanistic insight on how bioenergetic crisis coupled with oxidative stress leads to the demise of midbrain DA neurons. While PINK1 possesses an N-terminal mitochondrial targeting signaling, other PD-associated proteins, including α-synuclein, DJ-1, Parkin, and LRRK2, can partially associate to the OMM to regulate oxidative phosphorylation and energy production in neurons. Several landmark studies have shown evidence that PINK1 and DJ-1 are bona fide modulators of oxidative phosphorylation. For instance, various in vivo and cell culture studies have reported that a loss of function of PINK1 and DJ-1 contribute to a significant reduction in basal and maximal mitochondrial respiration in primary neurons, a disruption of mitochondrial membrane potential, and decreased complex-V dependent ATP synthesis [8, 135, 180, 181].

In Drosophila models, PINK1 regulates the electron flow of various complexes by modulating the proper assembly of complexes I and IV in the IMM [182]. Unlike PINK1, several in vivo and in vitro studies suggest that DJ-1 does not regulate the activity of ETC complexes nor impacts oxygen consumption rates. However, it was shown that DJ-1 regulates ATP synthesis via formation of a complex with ATP synthase by associating with the β subunit of the F1 sector to enhance the flow of protons through the channel of complex V [183-185]. Other studies have shown that DJ-1 regulates oxidative phosphorylation by regulating the assembly of complex I in the IMM [186]. In vivo, a significant reduction in mitochondrial respiration was observed in the striatum but not in the prefrontal cortex of 3-4-month-old PINK1-KO mice relative to wild-type mice. Similarly, a decrease in aconitase activity was observed, suggesting a deficiency in the TCA cycle as well [187]. In young, pre-symptomatic PINK1-KO rats, an in vivo PD model that develops motor symptoms and neurodegeneration of midbrain DA neurons, a substantial increase in oxygen consumption rates has been observed in the SN whereas the prefrontal cortex showed reduced mitochondrial respiration. These anatomical differences in phenotypes in the brain may be attributed to a compensatory elevation in mitochondrial respiration in the SN to help maintain a adequate level of ATP to sustain neuronal survival [188]. Interestingly, this increase in mitochondrial respiration (oxidative phosphorylation) was recapitulated in iPSC-derived neural cells expressing a PINK1 mutant (Q456X) which also exhibited increase proton leak; it is worth recognizing that this bioenergetic profile was distinct from iPSCs harboring LRRK2 mutations (G2019S or R1441C) which showed significantly decreased maximal respiration [189]. In addition, transient expression of PD-associated mutants of α-synuclein (e.g. AT53) leads to a decrease in basal and maximal mitochondrial respiration and a reduction in transmembrane potential, a mitochondrial pathology that is associated with mitochondrial fission and stalled mitochondrial trafficking in primary neurons, as discussed earlier [190]. An accumulation of the PD-associated mutant of α-synuclein oligomers has been observed to colocalize in mitochondria of SN neurons in α-synuclein overexpressing mice (A53T) as well as increased mitophagy and a reduction of complex I activity [191]. Showing convergence across in vitro and in vivo models of PD, this mitochondrial pathology induced by mutant α-synuclein is further exemplified in iPSC-derived DA neurons that express three copies of the SNCA gene or mutant A53T SCNA as evidenced by overt mitochondrial fragmentation, decreased baseline and maximal mitochondrial respiration and decreased transmembrane potential [192].

While there is a plethora of scientific evidence supporting a pathological role of PD-associated mutants of PINK1, DJ-1, and α-synuclein on mitochondrial pathology, several studies have provided evidence that LRRK2 is implicated in regulating oxidative phosphorylation and bioenergetics in different models of PD. The role of LRRK2 in regulating mitochondrial bioenergetics is exemplified in a study that showed that iPSCs derived from PD patients harboring a mutation that enhances the kinase activity of LRRK2 (G2019S), exhibited decreased basal and maximal oxygen consumption rates as well as enhanced the sensitivity of neurons to cell death induced by mitochondrial uncouplers such as valinomycin [193]. Given that familial PD affects non-neuronal tissues in humans, the use of PD patient-derived fibroblasts has provided potential prognostic value and valuable insight to understand how PD-associated mutations in PINK1, LRRK2 and DJ-1 contribute to mitochondrial dysfunction and bioenergetics alterations in humans [133, 194-196]. A study performed in primary human fibroblasts derived from PD patients harboring PD associated mutations in LRRK2 (G2019S) exhibited alterations in mitochondrial respiration, reduced mitochondrial-derived ATP levels and fragmented mitochondria [194, 195], presumably by directly associating with the mitochondrial fission regulator Drp1. Another study showed that patient-derived dermal fibroblasts and iPSCs harboring PD-associated mutations in LRRK2 exhibited a significant decrease in maximal reserve capacity and reduced complex I activity, a mitochondrial pathology that is reversed when inorganic nitrite is administered to activate the antioxidant Nrf2 pathway [197]. In other studies, iPSCs that harbor the G2019S mutation in LRRK2 showed significantly reduced antioxidant responses including suppressed Nrf2 signaling and a concomitant increase in KEAP1 which hampers this prosurvival antioxidant response pathway. As in patient-derived fibroblast, iPSCs expressing LRRK2- G2019S showed decreased maximal oxygen consumption rates and spare respiratory capacity as well as elevated mitochondrial-derived superoxide levels [198]. Overall, both cell culture and in vivo genetic models of PD suggest that PINK1, LRRK2 and DJ-1 interact at the OMM to form a unique trimeric complex to participate in a linear or parallel neuroprotective signaling pathway to regulate brain bioenergetics by regulating the activities and formation of supercomplexes of several ETC proteins or by directly modulating complex V-dependent ATP synthesis (Fig. 3). Finally, while there may be differences between the mechanism of mitochondrial dysfunction observed across familial models of PD in transgenic or knockout mice or iPSC-derived neurons (e.g. LRRK2-G2019S, PINK1-KO, DJ-1-KO), there is considerable agreement that a significant decrease in complex I activity, which is not caused by differences in protein levels, has been consistently observed in postmortem brain tissue from PD humans and contributes to decreased mitochondrial-derived ATP production and enhanced oxidative stress in PD [199].

### Mitochondrial Ca^2+^ Homeostasis

Ca^2+^ are universal secondary messengers that relay extracellular and intracellular stimuli in the cell resulting in a distinct physiological effect. Compared to the cytosol, Ca^2+^ is found in extremely high concentration (20,000-fold) in the extracellular space, generating a concentration gradient and allowing the cell to react to external and internal stimuli with precise spatiotemporal resolution. In addition to the high concentration gradient across the plasma membrane, the presence of intracellular Ca^2+^ stores such as the endoplasmic/sarcoplasmic reticulum and mitochondria can localize the Ca^2+^ signaling to microdomains in the cell for a targeted effect. Ca^2+^ signaling is indispensable for fundamental physiological processes from fertilization of oocytes to cell death modulation via the activation of the permeability transition pore. Ca^2+^ plays a vital role in the functioning of classical mitochondrial processes such as ATP production, ROS generation, and apoptosis. However, excessive mitochondrial Ca^2+^ levels can overwhelm mitochondrial Ca^2+^ buffering mechanisms and cause mitochondrial dysfunction. While mitochondrial dysfunction has been attributed to pathology in familial and idiopathic cases of PD, the pathological mechanisms underlying the neuronal loss in SN of PD patients remain unclear. DA neurons in SN are intrinsically distinct from other neuronal populations as they are autonomously active, producing basal oscillating action potentials regularly (2-4 Hz) in the absence of synaptic input. SN dopamine neurons employ L-type Ca^2+^ channels in lieu of typical (canonical) sodium channels to maintain the pace-making activity [200].

**Figure 4.**
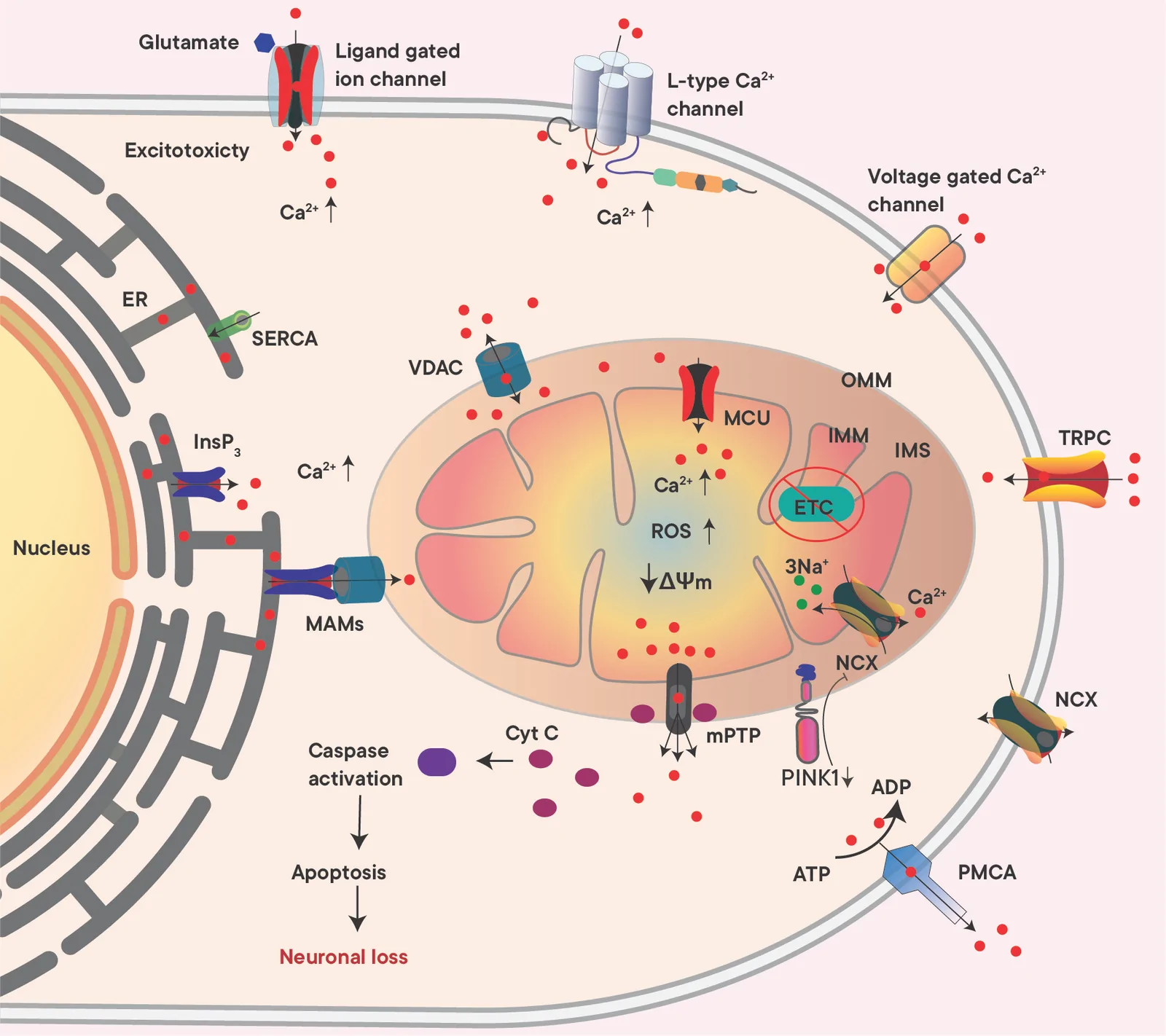
**Mitochondrial Ca^2+^ overload and Parkinson’s disease pathology**. In neurons, Ca^2+^ enters the cell through nicotinic acetylcholine receptors (nAChRs), voltage-gated Ca^2+^ channels (VGCCs), N-methyl-D-aspartate (NMDA) glutamate receptors, and transient receptor potential canonical (TRPC) channels. Additionally, Ca^2+^ release from internal stores like the endoplasmic reticulum (ER) is regulated by inositol trisphosphate receptors (IP3Rs). Moreover, Ca^2+^ is removed from the cytosol through the action of plasma membrane Ca^2+^ ATPases (PMCAs), sodium- Ca^2+^ exchangers (NCXs), and sarco/endoplasmic reticulum Ca^2+^ ATPases (SERCAs). Pacemaking activity of DA neurons is sustained by Ca^2+^ influx through L-type voltage-gated Ca^2+^ channels (VGCCs) and contributes to enhanced levels of cytosolic Ca^2+.^ In addition to the ER, mitochondria are involved in Ca^2+^ sequestration through SERCA pumps. Mitochondria acquire Ca^2+^ via mitochondrial Ca^2+^ uniporter (MCU), and Ca^2+^ efflux is performed by Na+/Ca^2+^ exchanger (NCX). In PD, pathological conditions alter the ability of mitochondria to sequester Ca^2+^ efficiently, leading to mitochondrial Ca^2+^ overload. Excessive Ca^2+^ taken up by mitochondria could lead to the activation of the mitochondrial permeability transition pore (mPTP), leading to a reduction in the transmembrane potential and increased ROS generation, which damages the ETC complexes and alters oxidative phosphorylation. In addition, mitochondrial swelling is evident, which induces the release of apoptotic factors such as cytochrome c from the mitochondrion leading to the downstream activation of caspases and eventual loss of neurons by apoptosis.

Although, pace-making activity ensures DA signaling, it has been argued that pace-making activity exposes the DA neurons in SNpc to higher levels of Ca^2+^. While a modest, transient increase in Ca^2+^ is critical to upregulate the rate of mitochondrial-dependent ATP synthesis, mitochondrial Ca^2+^ overload could compromise the sequestration process and make mitochondria susceptible to oxidative stress by activating the permeability transition pore, perturbs mitochondrial respiration, disrupts mitochondrial membrane potential, thus causing cytochrome C release and subsequent neuronal death via activation of apoptosis (Fig. 4). Advances in Ca^2+^ imaging and genetic characterization have enabled better understanding of the molecular mechanisms of mitochondrial Ca^2+^ handling. Cytosolic Ca^2+^ must pass through two membrane systems with differential gating mechanisms to reach the mitochondrial matrix. The OMM is relatively highly permeable to Ca^2+^ through pores comprised of voltage-dependent anion-selective channel proteins (VDACs). The Ca^2+^ passes through the IMM predominantly through a highly regulated mitochondrial Ca^2+^ uniporter (MCU) complex. The presence of MCU had been described several decades ago [201]; however, the molecular and structural characteristics of the uniporter and its regulators were discovered much later [202]. The development of a database of genes that encode for mitochondrial-localized/associated proteins led to the identification of coiled-coil domain-containing protein 109A (CCDC109A) as MCU [203]. This resulted in identifying MCU as the ruthenium red sensitive channel forming protein, predominantly responsible for Ca^2+^ influx into mitochondria. MCU oligomerizes in the IMM as a 485 kDa protein complex composed of subunits including EF-hand-containing Ca^2+^-binding proteins including the mitochondrial Ca^2+^ uptake 1 (MICU1), MICU2, and the pore-forming subunit MCU [204]. Regulators of MCU function have also been reported, including MCUR1, EMRE and miR-25 [205, 206]. One of the distinctive properties of MCU is its very low affinity for Ca^2+^ (20-30 μM under physiological conditions) [207], revealing that Ca^2+^ flux into mitochondria through MCU takes place only when the cytosolic concentration is in the range of 5-10 μM. This high concentration is quite rare in the cytosol except in certain micro domains juxtaposed to the ER and plasma membrane, where Ca^2+^ release takes place [208]. In order to alleviate mitochondrial Ca^2+^ levels and prevent mitochondrial swelling and subsequent dysfunction/fission, Ca^2+^ extrusion is mediated through the Na^+^/Ca^2+^ exchanger (NCX).

Disruption of Ca^2+^ homeostasis is a common "pathological" denominator in several neurodegenerative disorders including PD [209]. This concept is supported by several reports that show that several proteins encoded by PD-associated genes, including PINK1, α-synuclein and DJ-1 regulate mitochondrial Ca^2+^ handling. DJ-1 is a mitochondrial redox-sensitive sensor, with a multitude of other functions, that protects DA neurons by facilitating Ca^2+^ -mediated mitochondrial uncoupling and ROS generation during physiological pacemaking activity. Although the role of α- synuclein is not clear, there are reports that show that an overt and sustained intracellular Ca^2+^ increase elicits α-synuclein aggregation, which in turn promotes intracellular Ca^2+^ increase. However, there are some reports demonstrating the role of mitochondrial Ca^2+^ dyshomeostasis caused by a loss of endogenous PINK1 in neuroblastoma B103 cells [210]. The physiological role of PINK1 in modulating mitochondrial Ca^2+^ handling was observed in an experiment where co-expression of mutant, but not wild type (WT), worsened mitochondrial dysfunction as evident by an overt increase in mitochondrial Ca^2+^, a reduction in mitochondrial membrane potential and ATP synthesis. However, blocking the mitochondrial influx led to restoration of original phenotype in zebrafish [10, 211]. In another report, it was proposed that a lack of endogenous PINK1 caused a disruption of mitochondrial Ca^2+^ efflux by altering the activity of the mitochondrial Na^+^/Ca^2+^ exchanger and thereby inducing mitochondrial Ca^2+^ overload as evidenced in cultured mouse primary neurons and SH-SY5Y cells [212]. Therefore, the molecular identification of MCU has enabled the study of the exact role and mechanism of mitochondrial Ca^2+^ in PD (Fig. 4). Alterations in Ca^2+^ handling via the MCU have been observed in multiple recent studies performed in iPSC-derived DA neurons from PD patients harboring mutations in LRRK2, GBA1, Parkin SNCA and GBA1 as evidenced by an increase in cytosolic Ca^2+^ [213, 214]. Furthermore, in patient derived fibroblasts expressing a mutants of Parkin, an increase in ER-mitochondrial Ca^+2^ which was associated by an increase in the uptake of mitochondrial Ca^2+^ as evidenced by mitochondrial Ca^2+^ indicators [215].

## Considerations regarding technical limitations across models of PD

When studying mitochondrial-related signaling pathways in PD, it is worth acknowledging some of the limitations across models. Studies using murine-derived neurons and mouse models have shown changes in neurochemistry in PD, such as mitochondrial dynamics, function, biogenesis, and oxidative stress. These findings are congruent with research on postmortem brain tissue from PD patients and iPSC-derived neurons. However, genetic mouse models of PD (PINK1-KO, Parkin-KO, DJ-1-KO) have notable limitations, as these models do not recapitulate overt neurodegeneration of SN or striatal neurons compared to chemical models of PD or genetic rat models of PD [216-218]. One major advantage regarding the use of human cancer cell lines such as SK-N-SH or SH-SY5Y cells to chemically model PD (e.g., treatment with rotenone or MPP^+^) or for developing stable or inducible cell lines that knockdown PD genes is that they are of human origin which highly mirrors mitochondrial physiology compared to murine models. However, one major limitation is that many studies that have investigated mitochondrial-related signaling pathways in the context of PD have been done in naïve, undifferentiated neurons, a state that does not completely recapitulate neuronal physiology (e.g. lack of expression of Tyrosine Hydroxylase or DA Transporters) [219]. Additionally, the use of cancer cell lines, while convenient to use due to their immortalized nature which precludes the use of primary tissues (mouse or human-derived fibroblasts) may diverge in their physiology with additional passaging may become recalcitrant inducers of differentiation (retinoic acid or BDNF). Hence, the use of human iPSC-derived midbrain neurons is more relevant in terms of studying neurophysiology in midbrain DA neurons compared to murine-derived primary neurons; however, there is significant variability in neuron yield (70-85%), requires a significant amount of resources (e.g. requires transduction with four adeno-associated viruses and differentiation chemicals) and maintenance and the use of isogenic controls, which can be laborious. Hence the use of iPSC-derived neurons, while they can provide a significant amount of insights, can hamper the collection of data in a high yield manner. Overall, given the several aforementioned limitations across models of PD, this review emphasizes the need to use complementary models of PD (mouse or human in origin) to study the impact of mitophagy-related signaling pathways on the progression of PD [65].

## Therapeutic Implications

The current standard-of-care for treating motor symptoms of PD is the combination of Levodopa, to replenish and thereby increase the concentration of DA in the CNS of the patient and Carbidopa, to reduce the peripheral metabolic degradation of dopamine. However, as the disease progresses the wearing-off effect of levodopa is conspicuous and side effects including dyskinesia (uncontrolled muscular movements of the face and limbs) become prominent in the affected patient. Another approach is to treat PD patients with dopamine agonists such as pramipexole, bromocriptine or ropinirole, which mimic dopamine action on type 1 and 2 dopamine receptors. However, the therapeutic effect of dopamine agonists on treating PD symptoms is much weaker than levodopa. Monoamine oxidase B (MAOB) inhibitors supplemented with levodopa are also used to reduce PD symptoms. MAOB inhibitors such as Selegiline prevent the catabolism of dopamine to elevate the concentration of dopamine in the brain [220-224]. Finally, surgical interventions including deep brain stimulation (DBS) supplemented with levodopa therapy has been found to be effective in advanced PD patients [225]. The limitations in the present treatment strategies including side effects and diminished efficacy with progression of PD prompt the development of disease modifying therapies that can slow, stop or reverse the disease.

Recently, given that disruption in mitochondrial function plays a pathological role in PD, a large interest in the expansion of the pharmacological armamentarium for treating PD includes discovering and characterizing natural and synthetic compounds that can restore several aspects of mitochondrial function, including mitophagy, and biogenesis in PD patients. Studies in cell and animal models of PD acknowledge the beneficial effect of therapeutic interventions targeting mitochondrial function. While preclinical studies that assessed the therapeutic potential of antioxidants have demonstrated promising outcomes in reversing motor symptoms and delaying neurodegeneration of SN neurons in in vivo chemical models of PD, antioxidants such as glutathione and mitochondrially-directed coenzyme Q (MitoQ) have failed in clinical studies as they were unable to significantly improve clinical outcomes in PD patients [226, 227]. However, there are multiple clinical trial studies being performed to assess the beneficial effect of elevating mitochondrial function in PD patients by employing mitochondrial-targeted antioxidants and metabolites to alleviate mitochondrial dysfunction and thereby improve patient clinical outcomes (Table 1). For instance, in a placebo-controlled, double-blinded, randomized, phase 2 study (NCT03840005), the effect of Ursodeoxycholic acid (UDCA) on mitochondrial bioenergetics was found to be well tolerated in control and PD patients and evidenced increased ATP hydrolysis along with improved motor symptoms. Further investigation into the disease-modifying effects of UDCA is necessary [228]. UDCA has been shown to stimulate key mitochondrial functions in fibroblasts of PD patients. In a different study, EPI-589 or (R)-troloxamide quinone is claimed to stimulate glutathione (GSH) levels and curb oxidative stress. Clinical trials have recently been conducted by PTC Therapeutics in idiopathic PD patients to analyze the potential mitoprotective effects of EPI-589 (NCT02462603) on peripheral blood and brain biomarkers along with assessments of clinical symptoms in idiopathic PD patients. Another mitochondrial-targeted drug being studied for therapeutic intervention in PD patients is CNM-Au8 developed by Clene Nanomedicine. CNM-Au8 is a concentrated suspension of gold nanocrystals and is reported to enhance redox coenzyme nicotine adenine dinucleotide (NAD^+^) levels, ATP levels and reduce the level of ROS. A phase 2 clinical trial study was completed with CNM-Au8 (NCT03815916) in healthy and PD patients to assay oxidized to reduced forms of NAD^+^ through non-invasive magnetic resonance spectroscopy (MRS). This study showed enhanced brain NAD^+^/NADH ratio following CNM-Au8 treatment [229]. In the same line, the effect and dosage of NAD^+^ precursor nicotinamide riboside (NR) supplementation is being investigated in clinical trials (NCT03568968, NCT05589766). A wide range of potential antioxidant therapies are under clinical trials as well. Idebenone (CV-2619) enhances mitochondrial respiration and a multi-center, randomized, double-blind, placebo-controlled Phase 4 study (NCT03727295) is being carried out in early PD patients. The pathogenic mutation in LRRK2 causing heightened kinase activity is linked with impaired mitochondrial function and is prevalent among both sporadic and inherited forms of PD. Inhibitors of LRRK2 such as DNL201 and DNL151 have been studied in large randomized, double-blinded placebo-controlled Phase 1b studies in PD (NCT03710707, NCT04056689) and have shown promise in modifying lysosomal dysfunction in PD [230]. In conjunction with mitochondrial implications, PD has been suggested as a metabolic disease. Metformin is a commonly prescribed medication for the treatment of type 2 diabetes. It acts by increasing the sensitivity of liver and peripheral tissue to insulin in addition to reducing hepatic glucose output. More specifically, Metformin has been shown to enhance AMPK activity, which reduces oxidative stress and enhances mitochondrial biogenesis to promote neuroprotection. In PD models, treatment with Metformin showed reduced DA neuron loss and α-synuclein phosphorylation as well as reduced mRNA expression of pro- and anti- inflammatory signals [231-234]. A phase 2 clinical trial (NCT05781711) is investigating the effects of metformin in PD patients. A list of the aforementioned compounds in the pipeline, its target and progress in clinical trials is shown in Table 1. These and other compounds, such as the pharmacological activator of PINK1, kinetin, hold promise in modifying the pathology of PD and offer hope for treatment to halt and even reverse the neurodegeneration witnessed in PD.

**Table 1:** Parkinson’s disease therapeutics.

Therapeutic	Clinical Trial Number	Stage	Molecular or Mitochondrial Target
Ursodeoxycholic acid (UDCA)	NCT03840005	Phase 2	Enhance mitochondrial bioenergetics
EPI-589	NCT02462603	Phase 2	Mitigate oxidative stress by stimulating glutathione
CNM-Au8	NCT03815916	Phase 2	Increase ATP and reduce ROS through enhanced NAD^+^ levels
Nicotinamide riboside (NR)	NCT03568968	Phase 3	Enhance NAD+ levels through treatment with its precursor
	NCT05589766	Phase 2	
Idebenone (CV-2619)	NCT03727295	Phase 4	Enhance mitochondrial respiration by enhancing antioxidant activity
DNL201	NCT03710707	Phase 1	Mitigate lysosomal dysfunction by inhibiting LRRK2
DNL151	NCT04056689	Phase 1	
Metformin	NCT05781711	Phase 2	Enhance insulin sensitivity, mitochondrial function, and reduce neuroinflammation through AMPK activation

Many clinical trials are being performed aimed at modifying disease pathology at the mitochondrial level. The clinical trials vary in stage as they progress through the approval process. Their specificity ranges from direct protein inhibitors to broad bioenergetic metabolic precursors. Regardless of specificity, these emerging therapeutics provide optimism in the generation of disease-modifying therapies for PD.

## Conclusion

Here we provided a comprehensive overview of scientific and clinical evidence that supports the concept that mitochondrial dysfunction plays a prominent role in the development and progression of PD. This evidence is supported by postmortem brain tissue, cell culture and in vivo models of PD that show that exposure to PD toxins that affect mitochondrial function and expression of PD-associated mutants of DJ-1, PINK1, Parkin, LRRK2, and α-synuclein, induce converging mitochondrial pathology including impaired mitochondrial respiration, mitochondrial shape/structure, mitochondrial trafficking, regenerative capacity, biogenesis and turnover leading to chronic oxidative stress and activation of apoptotic pathways to promote neurodegeneration of SN neurons. The myriads of genetic studies of PD provides strong evidence that a neuroprotective signaling axis, conformed by DJ-1, PINK1, LRRK2, and Parkin, can converge at the mitochondrion or in parallel to support mitochondria function and quality control in healthy neurons. In addition, while a multitude of standard-of-care compounds are available to treat motor symptoms, these are no disease-modifying therapies. However, the current therapeutic pipeline intended to reverse mitochondrial dysfunction, while increasing mitophagy and mitochondrial biogenesis (Table 1), offers hope for the emergence of FDA-approved compounds that can be effective as disease-modifying therapeutics.
